# A DNA vaccine encoding the viral hemorrhagic septicemia virus genotype IVb glycoprotein confers protection in muskellunge (*Esox masquinongy*), rainbow trout (*Oncorhynchus mykiss*), brown trout (*Salmo trutta*), and lake trout (*Salvelinus namaycush*)

**DOI:** 10.1186/s12985-016-0662-8

**Published:** 2016-12-02

**Authors:** Isaac F. Standish, Elena V. Millard, Travis O. Brenden, Mohamed Faisal

**Affiliations:** 1 Department of Pathobiology and Diagnostic Investigation, College of Veterinary Medicine, Michigan State University, East Lansing, MI 48824 USA; 2 Department of Fisheries and Wildlife, College of Agriculture and Natural Resources, Michigan State University, East Lansing, MI 48824 USA

**Keywords:** DNA vaccine, Viral hemorrhagic septicemia virus, Fish

## Abstract

**Background:**

The viral hemorrhagic septicemia virus (VHSV) is one of the most serious fish pathogens. In 2003, a novel sublineage (genotype IVb) of this deadly virus emerged in the Great Lakes basin causing serious fish kills. We have previously demonstrated that a DNA plasmid (pcDNA), containing a cytomegalovirus (CMV) promoter and the viral hemorrhagic septicemia virus (VHSV) genotype IVb glycoprotein (G) gene insert (designated pVHSivb-G) confers moderate protection in muskellunge (*Esox masquinongy*), a highly susceptible species upon challenge. In order to achieve optimal protection, we investigated a number of factors including the incubation time [i.e. the number of degree days (° days)] before challenge, and viral challenge dose and route. Additionally, we tested if pVHSivb-G provides protection against VHSV-IVb to less susceptible salmonids such as rainbow trout (*Oncorhynchus mykiss*), brown trout (*Salmo trutta*) and lake trout (*Salvelinus namaycush*).

**Results:**

An increase in the period lapsed between vaccination and challenge to 1880° days resulted in 95% relative percent protection (RPS) in muskellunge following a single administration of the pVHSivb-G plasmid and viral challenge. An RPS of 100% for muskellunge was achieved with a longer incubation period (2400° days) and in conjunction with a booster dose of the plasmid. The pVHSivb-G vaccine also elicited significant protection in all three salmonid species, reaching 100% RPS in lake trout following an incubation period of 1001° days prior to viral challenge. Vaccination with pVHSivb-G was also associated with the development of significant levels of circulating VHSV-binding antibodies in muskellunge as measured by indirect ELISA, which reached peak levels 6–7 weeks post-vaccination. Viral shedding in vaccinated survivors was minimal and of transient nature.

**Conclusions:**

The study shows that the pVHSivb-G plasmid can elicit a protective response against the wild virus strain in a range of species important in recreational and commercial Great Lakes fisheries.

**Electronic supplementary material:**

The online version of this article (doi:10.1186/s12985-016-0662-8) contains supplementary material, which is available to authorized users.

## Background

The emergence of a novel *Novirhadovirus*, viral hemorrhagic septicemia virus (VHSV) genotype (IVb) in the Laurentian Great Lakes basin of North America alarmed fishery managers and researchers to the consequences its spread could bring to cultured and wild fish populations [1, 2]. This VHSV sublineage has an unusually wide host range, infecting 28 fish species, with muskellunge (*Esox masquinongy*) being the most susceptible species documented to date [3–6]. In the Great Lakes basin, numerous state and federal fish hatcheries are involved in propagation and stocking of a variety of fish species whose populations have been drastically suppressed from historical levels and in some cases are at risk of being endangered [7]. Of these fish stocks, millions of muskellunge and salmonid fry and fingerlings are annually propagated and released throughout the basin to support recreational and commercial fisheries. Salmon, lake sturgeon (*Acipenser fulvescens*), and muskellunge propagation relies on the collection of gametes from wild fish, while other programs [(e.g., rainbow trout (*Oncorhynchus mykiss*), brown trout (*Salmo trutta*), and lake trout (*Salvelinus namaycush*)] raise captive broodstocks [8]. Field observations and experimental studies have clearly demonstrated differing species susceptibility to VHSV-IVb, with salmonids exhibiting low to moderate susceptibility [2, 4, 5]. While the risks of VHSV-IVb are highest to the basin-wide muskellunge rehabilitation program, there are major concerns that salmonids may also aid in virus dissemination. This is particularly true since VHSV-IVb has been previously detected in apparently healthy salmonid species and gametes (reviewed in Faisal et al. [2]). To date, stringent biosecurity measures along with regulation of live fish transfer have successfully prevented VHSV-IVb from infecting hatchery populations. However, culture practices relying upon gamete collection from wild-fish and the stocking of progeny in public waters engender risks of VHSV-IVb introduction into hatcheries and consequently the potential of propagated fish to transmit VHSV-IVb upon stocking [9]. As a result, there is an urgent need to develop an efficacious VHSV vaccine that can protect multiple propagated fish species.

In a previous study, we explored the usefulness of a DNA-vaccine preparation, incorporating the VHSV-IVb glycoprotein (G) gene under the control of a human cytomegalovirus (CMV) promoter in protecting muskellunge upon challenge with the wild virus [10]. In that study, 539° days following an intramuscular administration (IM) of the plasmid, fish were immersion challenged with 10^5^ VHSV-IVb plaque forming units (pfu) mL^−1^. While administration of the G gene plasmid elicited the development of neutralizing antibodies, protection following challenge was moderate, achieving 45% relative percent survival (RPS) [10]. Encouraged by these results, the first aim of the present study was to optimize protective efficacy in vaccinated muskellunge by modifying a number of factors in the vaccination regimen such as time between vaccination and challenge, virus challenge dose, and the number of vaccine doses administered. We further investigated if vaccinated fish that survive infection were able to mount a humoral immune response and whether survivors continued to harbor and shed the VHSV-IVb. The second aim of the study was to determine if this same vaccine preparation could also be used to protect three representative genera of salmonids found in the Great Lakes basin: rainbow trout, brown trout and lake trout.

## Methods

### Fish

Two groups of muskellunge were used in this study. The first group of muskellunge, used in the first two muskellunge trials (MUS-1 and MUS-2), was obtained 16 weeks post-hatch [average 14.2 cm (*SD* = 1.4) fork length, 11.9 g (*SD* = 3.8)] from Chautauqua State Fish Hatchery (New York Department of Environmental Conservation, Chautauqua, NY). The second group of muskellunge, used in the third trial (MUS-3), was obtained 14 weeks post-hatch [average 15.6 cm (*SD* = 0.8) fork length, 16.0 g (*SD* = 1.1)] from Wolf Lake State Fish Hatchery [Michigan Department of Natural Resources (MDNR), Mattawan, MI)]. Rainbow trout (land-locked Eagle Lake morphotype) were obtained 12 weeks post-hatch [average of 3.6 cm (*SD* = 0.4) fork length, 0.7 g (*SD* = 0.3)] from the Oden State Fish Hatchery (MDNR, Alanson, MI). Brown trout (Gilchrest morphotype) were obtained 16 weeks post-hatch [average 7.5 cm (*SD* = 1.1) fork length, 4.2 g (*SD* = 1.7)] also from the Oden State Fish Hatchery. Lake trout (Lake Superior strain) were obtained 18 weeks post-hatch [average 17.6 cm (*SD* = 1.4) fork length, 24.1 g (*SD* = 6.5)] from Marquette State Fish Hatchery (MDNR, Marquette, MI).

All fish were certified to be free of important diseases according to World Organization for Animal Health (OIE) testing guidelines [11]. Fish were acclimated in 500-L circular fiberglass tanks in a continuous flow-through system with facility-chilled well water (11 °C) and supplemental aeration. Vaccination and experimental challenges were performed at the Michigan State University Research Containment Facility (East Lansing, MI). Muskellunge were fed live fathead minnows (*Pimephales promelas*) obtained from Anderson Farms Inc. (Lonoke, AR) that were certified free of important diseases according to OIE guidelines. An additional 60 fathead minnows were necropsied and underwent additional testing according to the American Fisheries Society guidelines [12]. Rainbow trout and lake trout were fed Skretting dry pellets (Skretting USA; Tooele, UT), while brown trout were fed Nelson’s Silver Cup trout feed (Harrietta Hills, Harrietta, MI). All fish were fed ad libitum throughout the study except for muskellunge during the 1st week post viral challenge when minnows were withheld. Two weeks prior to immunization, fish were randomly distributed among 72-L polyethylene flow-through tanks (Pentair Aquatic Eco-Systems, Apopka, FL) with supplemental aeration.

### Virus and cell culture

The VHSV genotype IVb isolate used in this study was the Great Lakes index strain MI03 [1]. The isolate has been maintained by continuous subculture in the cell line *epithelioma papulosum cyprini* (EPC). The EPC cell line was maintained and subcultured in 150-cm^2^ tissue culture flasks (Corning Inc., Corning, NY) at 25 °C using a basal media of Earle’s salt-based minimal essential medium (MEM) (Invitrogen Corp., Carlsbad, CA) and supplemented with 29.2 mg ml^−1^ L-glutamine (Invitrogen), penicillin (100 IU mL^−1^) (Invitrogen), streptomycin (0.1 mg mL^−1^) (Invitrogen), 10% fetal bovine serum (Gemini Bio Products, West Sacramento, CA), and sodium bicarbonate (7.5% w/v) (Sigma, St. Louis, MO). Viable viral concentrations were determined using plaque assay on EPC cell line using polyethylene glycol and a methylcellulose overlay [13, 14]. Virus was then aliquoted into cryogenic vials (Corning) for one time use and stored at − 80 °C.

### Construction of pVHSivb-G plasmid

The pcDNA_3.1 (+) is a commercially available vector containing the human CMV immediate-early promoter. The DNA vaccine construct containing the VHSV-IVb glycoprotein gene (designated pVHSivb-G, [10]) was modeled after successful DNA vaccines against VHSV genotype I [15, 16] and IHNV [17]. The construction and production of this plasmid were outsourced to Life Technologies (Carlsbad, CA). In brief, an *Eco*RI restriction site (G/AATTC) followed by a kozak consensus sequence terminating with the first amino acid of the complete MI03GL VHSV-IVb isolate glycoprotein gene (1524 bp) was synthesized; an *Xba*I restriction site (T/CTAGA) was then added following the 3′ termination codon. The assembled fragment was then digested using the described endonucleases and sub-cloned into the eukaryotic expression vector pcDNA 3.1 (+) (Invitrogen). The plasmid was transformed and propagated into K12 *Escherichia coli*. Sequencing confirmed the correct glycoprotein gene sequence and orientation. The pcDNA 3.1 (+) vector without the glycoprotein gene was similarly propagated and used as a negative (mock) control. Both plasmids were suspended in sterile PBS and stored at − 80 °C until use. The final products were designated pVHSivb-G and pcDNA (mock).

### Vaccination and challenge

Immediately prior to vaccination, all plasmid vectors were thawed and diluted to 10 μg in 100 μL sterile PBS. Randomly selected fish were anesthetized with 0.1 g L^−1^ of tricaine methanesulfonate (MS-222) (Western Chemical Inc., Ferndale, WA) and buffered with 0.3 g L^−1^ sodium bicarbonate. All fish were immunized IM with 10 μg of plasmid DNA in the left epaxial musculature. Nine trials took place which varied in number of °days (540 to 2400) within which fish were allowed to mount immune response prior to challenge. For all the trials using the three salmonid species, fish were allowed to mount an immune response for 1001° days prior to challenge. Additionally, booster doses were applied following 940° days in MUS-2 and MUS-3, and to a subset of individuals following 546° days in the salmonids trials. Fish were VHSV challenged using either intraperitoneal injection or immersion challenge. In trials using immersion challenge (see below), viral challenges were conducted in glass aquaria containing 32 L of chilled well water and supplemental external aeration. Fish were challenged via immersion in static water containing infectious virus for 60 min. All negative challenge controls were similarly challenged with minimal essential media. After 60 min, fish were returned to their respective tanks and monitored daily for morbidity and mortality for 28 days. Kidney, spleen and heart samples were collected from all mortalities, and inoculated onto EPC monolayers. After 14 days, supernatant was removed, frozen at − 80 °C, thawed at centrifuged at 2700 *g* for 15 min at 4 °C. Supernatant was then re-infected onto fresh EPC monolayer and incubated for 14 days before being examined for viral cytopathic effect (CPE). RNA from suspicious samples was then extracted using the QIAamp Viral RNA kit and following the manufacturer’s instructions. The presence of VHSV was confirmed using real-time reverse transcription polymerase chain reaction (RT-qPCR) assay specific for VHSV [18, 19].

### Muskellunge trials

In the first muskellunge trial (MUS-1), muskellunge were given a single 10 μg administration of either pVHSivb-G or pcDNA plasmids (*n* = 20 plasmid^−1^) and allowed to mount an immune response for 1880° days (24 weeks at 11 °C). Fish were divided into 2 tanks containing 10 fish each per preparation. An additional tank containing 10 muskellunge for each treatment was maintained throughout the study as non-infected controls. Experimental tanks were immersion challenged with 2 × 10^3^ pfu mL^−1^.

The second muskellunge trial (MUS-2) was identical to MUS-1 except after 940° days (12 weeks in 11 °C water), a booster dose of 10 μg of their respective plasmid was administered to each fish. Following a second period of 940° days, fish were immersion challenged with 2 × 10^3^ pfu mL^−1^ VHSV-IVb.

This third muskellunge trial (MUS-3) was nearly identical to that of MUS-2 except that fish were challenged 1460° days (an additional 520° days) following booster administration. Muskellunge therefore had a total of 2400° days to mount an immune response. Experimental tanks were challenged with 2 × 10^3^ pfu mL^−1^.

### Rainbow trout trials

The two rainbow trout trials (RBT-1, RBT-2) differed in conditions only in the VHSV challenge route. In both trials, fish were immunized IM with the pVHSivb-G or pcDNA plasmids (RBT-1: *n* = 39 [pVHSivb-G] and 29 [pcDNA]; RBT-2: *n* = 48 [pVHSivb-G] and 35 [pcDNA]). Fish from each treatment were returned to separate 72-L tanks and maintained throughout the study. After 546° days (6 weeks in 13 °C water), a portion of the pVHSivb-G vaccinated fish were administered a 10 μg booster dose (*n* = 13 [RBT-1] and 31 [RBT-2] respectively). Rainbow trout were then allowed to build an immune response for an additional 455° days prior to challenge (1001° days or 11 weeks at 13 °C post initial vaccination). Fish from RBT-1 were anesthetized and challenged by intra-peritoneal (IP) injection with 9.5 × 10^5^ pfu in 100 μL of sterile PBS. The negative control tank received sterile PBS. Fish from RBT-2 were challenged by immersion with 9.5 × 10^4^ pfu mL^−1^.

### Brown trout trials

Trials with brown trout (BNT-1, BNT-2) were the same as rainbow trout trials in respect to conditions and viral challenge. Brown trout were immunized IM with 10 μg of pVHSivb-G or pcDNA plasmids (BNT-1: *n* = 56 [pVHSivb-G] and 26 [pcDNA]; BNT-2: *n* = 50 [pVHSivb-G] and 28 [pcDNA]).. After 546° days (6 weeks in 13 °C water), (*n* = 18 [BNT-1] and 25 [BNT-2]), pVHSivb immunized brown trout received a 10 μg booster vaccine dose and were challenged 455° days later.

### Lake trout trials

For comparison purposes, the vaccination regimen in the two lake trout trials (LAT-1, LAT-2) was identical to the rainbow and brown trout trials. However, in lake trout trials the immersion challenge route was not performed, instead we used two IP challenge concentrations. Lake trout were immunized IM with the pVHSivb-G or pcDNA plasmids (both trials: *n* = 61 [pVHSivb-G] and 26 [pcDNA]). After 546° days (6 weeks in 13 °C water), (*n* = 27 for both LAT-1 and LAT-2), pVHSivb immunized lake trout received a 10 μg booster dose. Fish were challenged 455° days later. Fish in LAT-1 were IP challenged with 9.5 × 10^5^ pfu in 100 μL PBS while lake trout in LAT-2 were IP challenged with 4.75 × 10^6^ pfu in 500 μL of PBS (i.e., a five-fold greater VHSV concentration compared to LAT-1),.

### Detection of anti-VHSV antibodies in muskellunge sera using an indirect ELISA procedure

To examine the humoral response associated with the pVHSivb-G plasmid, blood samples were collected from 27 muskellunge from the Chautauqua State Fish Hatchery 2 weeks prior to immunization. Following collection, blood was stored at 4 °C for 2 h and centrifuged at 2700 *g* for 10 min at 4 °C. Serum was then aliquoted and stored at − 80 °C until analysis. Muskellunge then received an intramuscular vaccination of 10 μg of the pVHSivb-G preparation. Sera was collected and processed as described above every 2 weeks until 10 weeks (770° days) post vaccination. Half of the fish (*n* = 15) were then immersion challenged with 2 × 10^3^ pfu mL^−1^ as previously described. Sera were then collected from muskellunge 6, 12, 15, 18, 20, 30 and 34 weeks post challenge. All sera samples were analyzed using a newly developed and optimized muskellunge specific indirect ELISA to assess circulating anti-VHSV antibodies [20].

Prior to ELISA, serum was heat inactivated at 45 °C for 30 min. Serum was centrifuged at 2700 *g* for 10 min. at 4 °C immediately prior to dilution in a solution of 1% nonfat dried milk in PBS (dilution of PBS-5% NFDM, Sigma). Indirect ELISA took place in polystyrene microplates (96-well, Microlon®600 with chimney wells; Greiner Bio-One, Monroe, NC). Plates were sealed during all incubation periods (SealPlate®; Sigma) and washed 5 times following each incubation period unless otherwise stated using PBS containing 0.05% Tween 20 (PBS-T20; Sigma) in an automated microplate washer (BioTek, 4 L × 405™ plate washer; Winooski, VT).

Briefly, microtiter assay plates were coated with 100 μL well^−1^ of purified VHSV-IVb at 1 μg mL^−1^ and incubated overnight (14–16 h) at 4 °C in a humid chamber. After incubation, plates were washed and unbound sites were blocked with the addition of 430 μL well ^−1^ of PBS containing 5% NFDM (PBS-5%; Sigma) and incubation at 37 °C for 1 h. Heat inactivated and diluted test and control muskellunge sera was then added to duplicate wells at 100 μL well^−1^. After incubating at 25 °C for 1 h, plates were washed and 100 μL of 1:30,000 dilution of the 3B10 mouse anti-muskellunge-IgM mAb was added to all wells and again incubated at 25 °C for 1 h. Plates were washed and 100 μL of 1:4,000 dilution of a commercially available goat anti-mouse secondary horseradish peroxidase (HRP) conjugated antibody (Invitrogen) was added to each well and incubated at 25 °C for 1 h. Plates were developed by the addition of 100 uL of 0.4 mg mL^−1^ *o*-phenylenediamine (Sigma) in phosphate citrate buffer (Sigma) containing 3 mM hydrogen peroxide (Avantor Performance Materials Inc., Center Valley, PA). The reaction proceeded for 30 min at 25 °C in the dark and was stopped with the addition of 50 L of 3 M sulfuric acid (H_2_SO_4_; Avantor Performance Materials). The optical density (OD) was read at 490 _nm_ using a BioTek, ELx808™ plate reader (BioTek) and the Gen5 software (BioTek). The average value of blank wells was subtracted from test and control wells prior to analysis. A threshold of 0.163 was used as the basis for distinguishing between presence/absence of circulating antibodies [20].

### Assessment of VHSV shedding by vaccinated and challenged muskellunge

Surviving vaccinated muskellunge from the MUS-2 and MUS-3 trials were used to examine for the presence of VHSV shedding. Samples were collected beginning 4 weeks post challenge and every 4 weeks for 28 weeks. Shedding analysis was conducted using a slightly modified protocol of that described by Kim and Faisal [21]. Briefly, individual surviving fish were transferred to glass aquaria containing 4 L of static facility chilled well water containing supplemental aeration. After 120 min, water was mixed and a 50 mL water sample was taken and fish were placed back into their original tanks. Samples were stored at 4 °C until processing within 24 h. For processing, samples were vortexed and centrifuged at 2700 *g*, at 4 °C for 10 min. After centrifugation, a viral plaque assay (VPA) was conducted as previously described. After 6 days, cell monolayers were stained with crystal violet (Sigma) and 18% formaldehyde (Avantor Performance Materials Inc.).

### Statistical analysis

For muskellunge trials (MUS-1,2,3) Kaplan-Meier survival estimates and mean time to death accounting for right censoring of survival data (i.e., mortality did not occur during the 28 day monitoring period) were calculated for each tank of fish using PROC LIFETEST in SAS [22]. Cox proportional hazards frailty models with tank as a random effect to account for fish deaths within tanks not being independent were also calculated for all trials using PROC PHREG in SAS. In all trials, the cumulative mortality was averaged across replicate tanks when possible for each trial to calculate RPS for each treatment [23].

$$ \mathrm{R}\mathrm{P}\mathrm{S}=1-\left(\frac{\% cumulative mortality of vaccinated}{\% cumulative mortality of mock vaccinated}\right)\times 100 $$

In muskellunge trials (MUS-1,2,3), significant differences in cumulative mortality between or among treatments within a trial were tested using t-tests conducted using PROC TTEST in SAS. While, due to a lack of replication in the salmonid trials, statistical significance was analyzed using a conventional two-tailed χ^2^-test.

The humoral response in pVHSivb-G vaccinated and challenged muskellunge were initial examined using a paired *t*-test and subsequently analyzed using quantile regression, which allowed us to more fully examine the conditional relationship of the humoral response over time. Separate regression models were estimated for post vaccination and post challenge responses. Conditional 0.50, 0.75, and 0.90 quantile regressions were fit to each set of data. Quantile regression allows a more thorough examination of the conditional relationship between dependent and independent variables; for example, it can determine if the rate of change in more extreme values of the dependent variable is different than the change for central tendency values [24]. For model fitting, OD values were log_*e*_ transformed with time (i.e., post vaccination or post challenge times) up to its third power used as explanatory variables. The significance of explanatory variables (i.e., time, square of time, cube of time) was tested using likelihood ratio tests. If an explanatory variable was not statistically significant, it was excluded as an explanatory variable and the model was refit. 95% confidence intervals for parameter estimates from the quantile regression models were obtained by resampling with the number of resampling iterations set at 1,000. Quantile regression models were fit in SAS using PROC QUANTREG.

### Ethics statement

All experiments were conducted in accordance with the ethical guidelines defined by Michigan State University’s (MSU) Institutional Animal Care and Use Committee (AUF 03/14-047-00).

## Results

### Vaccine efficacy in muskellunge

For the MUS-1 trial, the two replicate tanks of muskellunge that received the pVHSivb-G plasmid experienced 0 and 10% cumulative mortality whereas the pcDNA plasmid replicates both experienced 100% mortality (Table 1), resulting in a mean RPS of 95%. The mean day of death for the pcDNA plasmid treatments was 11.0 (SE = 1.0) and 12.4 (SE = 1.3) whereas the mean day of death for the pVHSivb-G plasmid treatment was 16.0 (SE = NA) (mean day to death was only calculable for one tank and standard errors could not be calculated) (Table 1). The hazard ratio comparing the pVHSivb-G and pcDNA plasmids estimated from the Cox proportional hazards frailty model was 0.016 (95% confidence limits: 0.002–0.124), suggesting the pVHSivb-G treatment significantly reduced the hazard rate for muskellunge (Table 1). There was a statistically significant difference in cumulative mortality between the pVHSivb-G and mock treatments (*t*-test: *t* = −19.00, df = 2, *P*-value = 0.003). During necropsy, muskellunge exhibited characteristic clinical signs of acute VHSV infection including extensive petechial to ecchymotic hemorrhage throughout the musculature, liver, swim bladder and renal mesentery. Numerous fish exhibited severely pale gills, liver, and heart. VHSV-IVb was re-isolated from all of the mortalities on the EPC cell line.

**Table 1:** Summary of the three trials conducted using muskellunge (MUS-1, 2, 3)

	Fish	Cumulative mortality	Mean Cumulative Mortality	Mean day to death ± SE	HR (95%CI)	RPS	*P*-value
MUS-1							
pVHSivb-G	10	0%	5%	NA	0.016 (0.002–0.124)	95%	0.003
pVHSivb-G	10	10%					
pcDNA	10	100%	100%	11.7 ± 1.3		NA	NA
pcDNA	10	100%					
MUS-2							
pVHSivb-G	10	30%	15%	10.6 ± 0.4	0.063 (0.018–0.223)	85%	0.030
pVHSivb-G	10	0%					
pcDNA	10	100%	100%	9.9 ± 1.0		NA	NA
pcDNA	10	100%					
MUS-3							
pVHSivb-G	10	0%	0%	NA	NA	100%	<0.0001
pVHSivb-G	10	0%					
pcDNA	10	100%	100%	12.8 ± 1.8		NA	NA

Total number of fish challenged in each trial, mean cumulative % mortality, and mean days to death of each treatment. The table also includes mean day to death calculated using PROC LIFETEST in SAS [22]. A Cox proportional hazard model was also fitted using SAS to examine the hazard ratio (HR) between glycoprotein (G) gene and mock-vaccinated counterparts. The relative percent survival (RPS) was calculated between treatments as previously described [23]. The *P*-values are from t-tests testing survival differences between the treatments that were conducted using PROC TTEST in SAS

In the second muskellunge trial (MUS-2), replicate tanks of pVHSivb-G vaccinated muskellunge challenged 2400° days post-vaccination (PV, 940° days post booster administration) experienced 0 and 30% mortality (Table 1), with mean day of death of 10.6 (SE = 0.4) days (mean day of death could not be calculated for the other replicate since no mortalities occurred). Conversely, the pcDNA plasmid replicates experienced 100% mortality with mean day of death of 9.3 (SE = 0.9) and 10.4 (SE = 0.9) days, resulting in an RPS of 85%. The hazard ratio comparing the pVHSivb-G and pcDNA mock treatments was 0.063 (95% confidence limits: 0.018–0.223) (Table 1) suggesting the pVHSivb-G treatment significantly reduced the hazard rate for muskellunge. Cumulative mortality between the pVHSivb-G and pcDNA treatments was significantly different (*t*-test: *t* = 5.67, df = 2, *P*-value = 0.030). During necropsy, mortalities again exhibited distinct signs of acute VHSV-IVb infection.

In MUS-3, pVHSivb-G vaccinated muskellunge challenged 2400° days post-vaccination (PV, 940° days post booster administration) experienced 0% mortality while the pcDNA mock replicates experienced 100% mortality resulting in 100% RPS (Table 1). The mean day of death of the pcDNA replicates were 12.2 (SE = 1.7) and 13.4 (SE = 1.7) days. This was clearly a significant level of protection. However, the hazard ratio between the treatments could not be calculated because of the lack of variability in treatment results. Similarly, the lack of variability in the treatment results resulted in questionable *t*-test results with regards to differences in cumulative mortality (*t*-test: *t* = ∞, df = 2, *P*-value < 0.0001) (Table 1).

### Vaccine efficacy in salmonids

Mock-vaccinated rainbow trout in RBT-1 experienced 62% cumulative mortality following IP challenge (Table 2). In contrast, rainbow trout vaccinated with pVHSivb-G experienced only 30% cumulative mortality following a single administration and 15% mortality with two administrations. Significant protection was observed in G gene treatments, with 57% RPS (χ^2^ = 6.83, df = 1, *P*–value = 0.009) and 75% RPS (χ^2^ = 7.84, df = 1, *P* -value = 0.005) following one or two pVHSivb-G administration respectively (Table 2). In RBT-2, only 9% mortality of the mock-vaccinated fish was observed following challenge by immersion with the virus and none of the pVHSivb immunized trout died (Table 2). Due to low mortality experience in rainbow trout using immersion infection, significant protection was not observed. All mortalities exhibited clinical signs of VHSV infection including mild to moderate petechial to ecchymotic hemorrhage throughout the musculature, liver, swim bladder and renal mesentery. VHSV was re-isolated from all of the mortalities.

**Table 2:** Results of vaccination trials in three salmonid species

Treatment	Fish	Cumulative Mortality	RPS	χ^2^	*P*-value
RBT-1					
pcDNA	29	62.1%			
pVHSivb-G	26	26.9%	56.7%	6.83	0.009
pVHSivb-G (2 doses)	13	15.4%	75.2%	7.84	0.005
RBT-2					
pcDNA	35	8.6%			
pVHSivb-G	17	0.0%	100.0%	1.55	0.214
pVHSivb-G (2 doses)	31	0.0%	100.0%	2.78	0.095
BNT-1					
pcDNA	26	23.1%			
pVHSivb-G	38	5.3%	77.2%	4.48	0.034
pVHSivb-G (2 doses)	18	16.7%	27.8%	0.27	0.604
BNT-2					
pcDNA	28	10.7%			
pVHSivb-G	25	4.0%	62.7%	0.85	0.356
pVHSivb-G (2 doses)	25	4.0%	62.7%	0.85	0.356
LAT-1					
pcDNA	26	30.7%			
pVHSivb-G	34	0.0%	100.0%	12.07	0.0005
pVHSivb-G (2 doses)	27	0.0%	100.0%	9.79	0.0018
LAT-2					
pcDNA	26	34.6%			
pVHSivb-G	34	0.0%	100.0%	13.85	0.0002
pVHSivb-G (2 doses)	27	0.0%	100.0%	11.26	0.0008

Rainbow trout (RBT-1 and RBT-2), brown trout (BNT-1 and BNT-2) and lake trout (LAT-1 and LAT-2) vaccine trials. Table includes the number of fish in each treatment, the cumulative mortality and the relative percent survival (RPS) and the associated *P*-value calculated using a two-tailed χ^2^-test

In the first brown trout trial (BNT-1), mock-vaccinated brown trout experienced 23% cumulative mortality (Table 2) following IP challenge. Fish vaccinated with pVHSivb-G experienced 5% cumulative mortality with a single administration and 17% cumulative mortality following two administrations resulting in 77% and 28% RPS respectively. Protection was only significant in the treatment that received a single administration of the pVHSivb-G plasmid (χ^2^ = 4.48, df = 1, *P* -value = 0.034). In BNT-2, mock-vaccinated brown trout individuals experienced 11% cumulative mortality following immersion challenge (Table 2). The cumulative mortality of both the vaccinated treatments was 4% (63% RPS), resulting from only a single individual dying in both the treatments. Mortalities were again too low to determine significant protection. The virus was isolated from all mortalities, though brown trout exhibited milder clinical signs of VHSV infection including less petechial hemorrhage throughout the liver, swim bladder and renal mesentery.

In LAT-1, the mock-vaccinated lake trout experienced 31% cumulative mortality (Table 2). Meanwhile, not a single vaccinated individual from G gene treatments succumbed. Lake trout experienced significant protection with 100% RPS in both G gene treatments (χ^2^ = 12.07, df = 1, *P* -value = 0.0005 with a single administration), (χ^2^ = 9.79, df = 1, *P* -value = 0.0018 with two administrations). While, in the second lake trout trial (LAT-2), when IP challenged with a five-fold higher viral concentration, the mock-vaccinated treatment experienced 35% cumulative mortality while none of the vaccinated fish died (Table 2), resulting in 100% RPS and significant protection (χ^2^ = 13.85, df = 1, *P* -value = 0.0002 with a single administration, and χ^2^ = 11.26, df = 1, *P* -value = 0.0008 with two administrations). Lake trout mortalities exhibited clinical signs of VHSV infection as previously described and VHSV was isolated from all mortalities.

### Development of circulating anti-VHSV antibody response in pVHSivb-G vaccinated muskellunge

Prior to vaccination the OD levels in the 27 naïve muskellunge were 0.008 (*SD* = 0.008). Anti-VHSV antibodies did not appear to significantly increased until measured 28 days following vaccination, at which point the mean OD value had reached 0.248 (*SD* = 0.184) (paired *t*-test: *t* = 6.18, df = 23, *P*-value < 0.0001). Anti-VHSV antibody levels appeared to peak between 42 days [0.315 (*SD* = 0.313)] and 56 days [(0.3.72 (*SD* = 0.256)] post exposure. Though, to precisely predict peak antibody levels and the duration of the secondary anti-VHSV antibody response following challenge, quantile regression analysis was utilized.

**Fig. 1:**
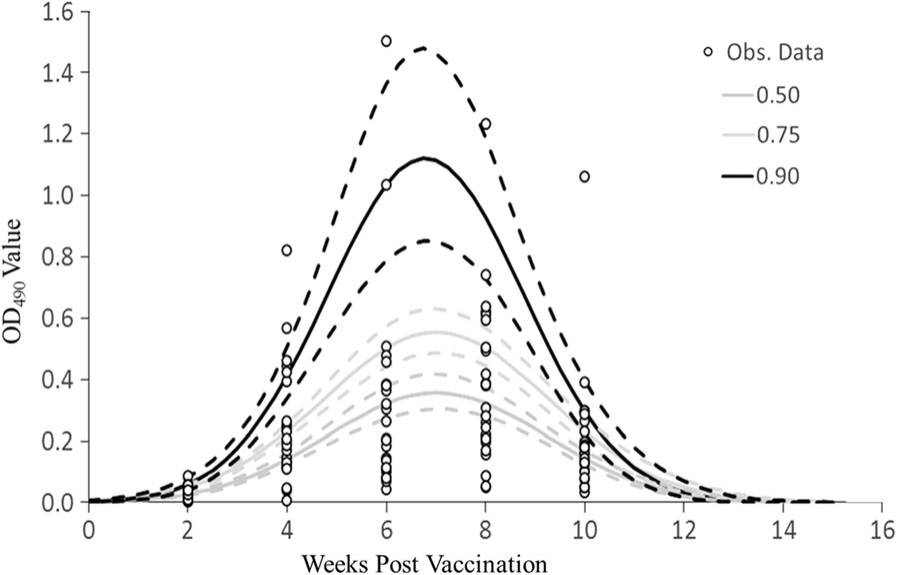
Levels of anti-VHSV antibodies in immunized muskellunge. Levels of circulating anti-VHSV antibodies of 27 muskellunge for 10 weeks vaccinated with 10 μg of the pVHSivb-G indicated by indirect ELISA optical density (OD_490_) values. The predicted relationships from 0.50, 0.75, and 0.90 quantile regression models was fit to the OD_490_ values as a function of time since vaccination. The dashed lines represent ±1 SE of the predicted relationships. The cut off value was estimated to be 0.163, above which a sample was considered positive

For all evaluated quantiles, the cube of time post-vaccination (PV) was not statistically significant by likelihood ratio testing and was dropped from the models (0.50: x^2^ = 3.30, df = 1, *P*-value = 0.069; 0.75: x^2^ = 1.47, df = 1, *P*-value = 0.226; 0.90: x^2^ = 3.80, df = 1, *P*-value = 0.051). However, the square of time PV was statistically significant (*P*-value <0.0001) for all models. The functional form of the quantile regression fitted relationships for the OD values was similar across the quantiles, and could perhaps best be characterized as bell-shaped (Fig. 1). The model corresponding to a 0.90 quantile had a somewhat steeper relationship then the other models as a consequence of a few outlying OD values at between 4 and 10 weeks post-challenge (Table 3). Based on predicted relationships from the fitted quantile regression models, OD values would increase to a value 0.163 by 3 to 4.25 weeks depending on the quantile (Fig. 1). Peak OD values would occur at around 7 weeks post vaccination, and would then decline back to 0.163 at around 10 to 11 weeks post vaccinations (Fig. 1).

**Table 3:** Parameter estimates of quantile regression models fit to log_*e*_ OD values

Quantile	Model output	Intercept	Time	Time × Time
Post-Vaccination				
0.5	Estimate	−6.037	1.427	−0.102
	standard error	0.532	0.193	0.015
	95% confidence limits	−2.107	1.045–1.809	−0.059
0.75	Estimate	−5.34	1.357	−0.097
	standard error	0.434	0.165	0.013
	95% confidence limits	−1.719	1.030–1.683	−0.052
0.9	Estimate	−5.82	1.749	−0.129
	standard error	0.845	0.323	0.025
	95% confidence limits	−3.349	1.109–2.389	−0.1
Post-Challenge				
0.5	Estimate	1.304	−0.069	NA
	standard error	0.187	0.103	NA
	95% confidence limits	0.931–1.678	−0.041	NA
0.75	Estimate	1.343	−0.048	NA
	standard error	0.224	0.016	NA
	95% confidence limits	0.896–1.791	−0.063	NA
0.9	Estimate	1.573	−0.055	NA
	standard error	0.317	0.021	NA
	95% confidence limits	0.938–2.208	−0.085	NA

Parameter estimates, standard errors, and 95% confidence limits from 0.50, 0.75, and 0.90 quantile regression models fit to log_*e*_ OD values post pVHSivb-G immunization (Post Vaccination) and post VHSV challenge (Post Challenge). The fitted regression models for post-vaccination (PV) included weeks PV (Time) and the square of weeks PV (Time × Time) as explanatory variables. The fitted regression models for post-challenge included weeks post-challenge (Time) as an explanatory variable

**Fig. 2:**
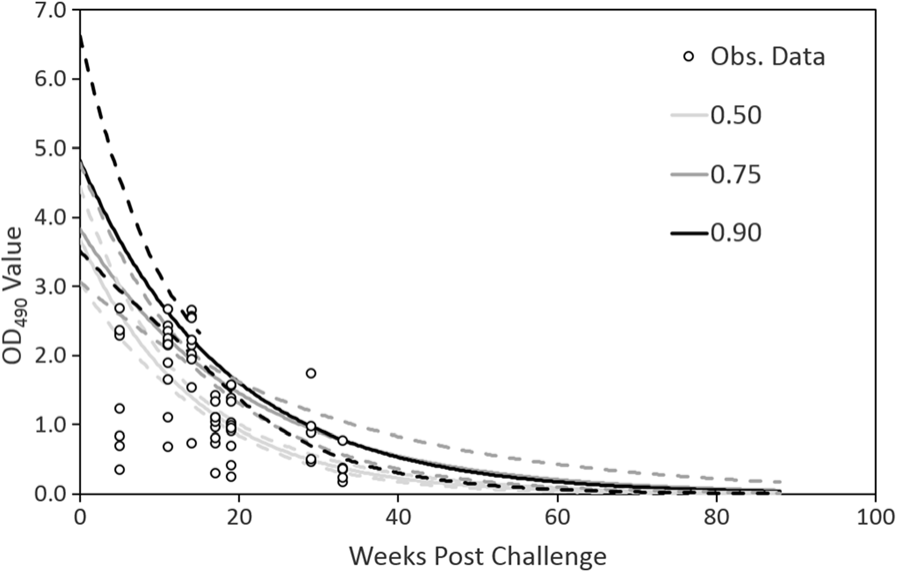
Levels of anti-VHSV antibodies in immunized muskellunge following VHSV challenge. Levels of circulating anti-VHSV antibodies of 15 muskellunge following vaccinated with 10 μg of the pVHSivb-G and VHSV immersion challenged [(2 × 10^3^ pfu mL^−1^) for 60 min] indicated by indirect ELISA optical density (OD_490_) values. Predicted relationships from 0.50, 0.75, and 0.90 quantile regression models fit to the OD values as a function of time since vaccination. The dashed lines represent ±1 SE of the predicted relationships. The cut off value was estimated to be 0.163, above which a sample was considered positive

For the quantile regression models fit to the post challenge data, the cube of time post-challenge was not statistically significant (0.50: x^2^ = 2.31, df = 1, *P*-value = 0.128; 0.75: x^2^ = 2.01, df = 1, *P*-value = 0.156; 0.90: x^2^ = 0.57, df = 1, *P*-value = 0.449). Similarly, the square of time post-challenge was not statistically significant (0.50: x^2^ = 2.09, df = 1, *P*-value = 0.148; 0.75: x^2^ = 2.17, df = 1, *P*-value = 0.141; 0.90: x^2^ = 1.44, df = 1, *P*-value = 0.231). Consequently, both terms were dropped from the models. Time post-challenge was statistically significant (*P*-value <0.005) for all models. The fitted relationships for each of the quantile regression models was suggestive of an exponential decline in OD values with respect to time (Fig. 2, Table 3), however, this was likely influence by the lack of observations for the 4 weeks immediately following challenge. Based on the predicted relationships from the regression models, OD values would not be expected to decline to less than the establish positive/negative threshold (0.163) until between weeks 45 and 65 post challenge (Fig. 2).

### Viral shedding in pVHSivb-G vaccinated survivors

**Fig. 3:**
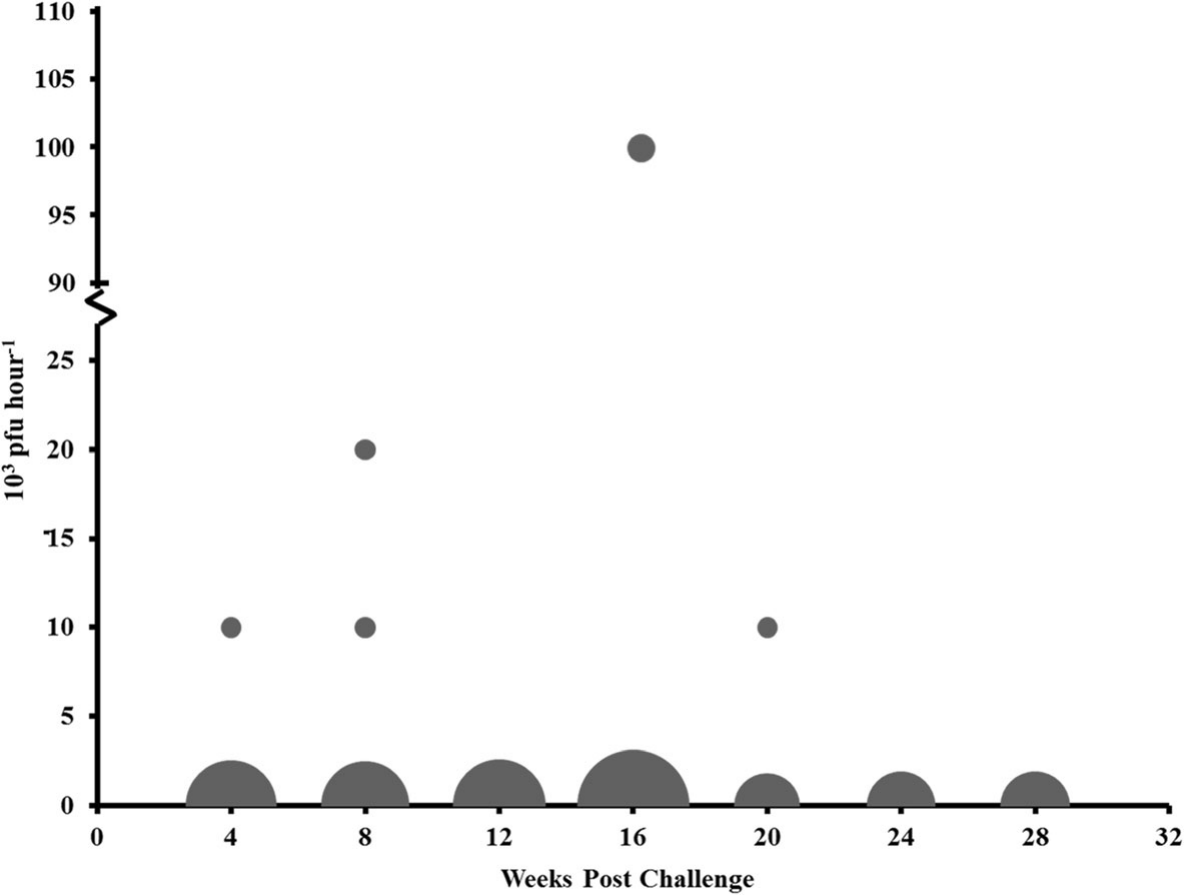
VHSV shedding in immunized muskellunge following shedding. Weighted bubble graph displaying the analysis of shedding in pVHSivb-G vaccinated muskellunge (*n* = 39) following a VHSV-IVb immersion challenge (2 × 10^3^ pfu mL^−1^ for 60 min). Viral plaque assays were conducted on water samples collected from individual surviving muskellunge every 4 weeks until 28 weeks post challenge

Shedding was assessed in pVHSivb-G vaccinated survivors from MUS-2 beginning 16 weeks post challenge and MUS-3 beginning 4 weeks post challenge using a quantitative viral plaque assay. Low level of shedding was detected from a few survivors, with one fish at 4 and 20 weeks post challenge (Fig. 3), and two fish 8 weeks post challenge shedding 10^4^ to 2.0 × 10^4^ pfu hour^−1^. Two fish exhibited the maximum shedding concentration (10^5^ pfu hour^−1^) 16 weeks post challenge. No shedding was detected after 20 weeks post challenge from any individual. Overall, viral shedding from vaccinated survivors appears minimal and transient.

## Discussion

Two decades of research have demonstrated that DNA-based vaccines can be highly effective for conferring protection against the European VHSV lineage (genotype Ia) in rainbow trout, a heavily cultured fish species [25–29]. Experimental DNA vaccine preparations have primarily incorporated the VHSV glycoprotein (G) gene under the control of a human cytomegalovirus (CMV) promoter [15, 28–33]. The G gene is targeted as the encoded protein is necessity for VHSV attachment and cellular internalization [30, 34–36]. Studies show that IM administrations of doses ranging from 1 μg [28, 29] to 10 μg [15] of a plasmid containing the VHSV glycoprotein (G) gene, followed by an incubation period of 400-880° days conferred 83–96% RPS in rainbow trout, when immersion challenged with 10^4^ to 3 × 10^6^ median tissue culture infectious dose (TCID_50_) mL^−1^ [15, 28, 29]. In our laboratory, when this protocol was used in muskellunge it did not protect over half of vaccinated fish upon challenge [10] and therefore this study was designed. When we increased the period that vaccinated muskellunge had to mount an immune response prior to challenge, this resulted in greater survivorship than the 45% RPS previously demonstrated. It seems that a longer time post-vaccination is needed to provide fish sufficient time to develop a protective immune response [37]. The fact that vaccinated fish need a relatively long time to develop a protective response dictates that the vaccine should be administered in their early life stages so that they are protected prior to their stocking in public waters. In muskellunge, the highest RPS (100%) was observed in MUS-3 when muskellunge received two administrations of the pVHSivb-G preparation, though a single administration in MUS-2 under otherwise similar conditions also resulted in a high RPS (95% RPS). It seems that administration of more than vaccine dose is not necessary to achieve protection.

In salmonids, we also demonstrated significant protection in each species following IP challenge, with RPS ranging from 27.8 to 100% in these trials. The 100% RPS observed in both the lake trout trials is comparable to the protection previously achieved with DNA preparations against VHSV-I and a similar novirhabdovirus, IHNV in rainbow trout [15, 29]. Though, salmonids are less susceptible to genotype IVb, and a high IP viral concentration was required to elicit mortality and demonstrate protection. If environmentally realistic concentrations were utilized [38], this would likely result in greater protection and survivorship then we have documented. RPS varied based on the administration of either one or two doses of the pVHSivb-G plasmid; in BNT-1, fish actually experienced greater protection following only one administration of the plasmid. Also lake trout experienced identical protection regardless of the number of plasmid administrations, providing further evidence that a single administration of the plasmid elicits sufficient protection.

We further demonstrate several novel aspects of post vaccine efficacy. For example, the indirect ELISA demonstrates that the pVHSivb-G plasmid elicited the development of a significant circulating VHSV-binding antibody response in muskellunge, which peaked around 7 weeks post vaccination. Though, in muskellunge from the previous study by Millard et al. [10], 7 weeks post vaccination corresponded with the initial development of a neutralizing antibody response, with only 1 of 12 vaccinated muskellunge exhibiting neutralizing anti-VHSV antibodies, though, 4 weeks later 60% of muskellunge exhibited neutralizing antibodies [10]. This discrepancy can be attributed to the fact the used indirect ELISA employed in this study detects all binding antibodies generated in response to the vaccine while neutralization antibody assay measure only one subset of these antibodies. Though, in this study, the incubation period and subsequent challenge we utilized with muskellunge did not appear to correspond with peak anti-VHSV binding antibody levels as indicated by the ELISA OD values. Peak binding antibodies levels may not correspond with peak protection; however, the magnitude of the humoral response does appear to indicate the development of an adaptive response and involving long lived plasma cells (LLPC) [39]. In studies done on rainbow trout using a non-microbial antigen, it has been demonstrated that antibodies produced 12–15 weeks following immunization have a much higher affinity to the antigen [40]. A similar phenomenon may be taking place in muskellunge thus explaining the significant protection despite the overall declining antibody levels. Further, in vaccinated muskellunge, following challenge, we were able to detect a vigorous secondary response. We were able to predict this response would to remain at detectable levels for upwards of 40–50 weeks and is suggestive a prolonged secondary immune response. The details of the kinetics of the primary and secondary humoral response provide a valuable input for future vaccination and release strategies if this preparation is approved for use in aquaculture. Needless to say, cell mediated immunity may also play a major role in protection of vaccinated fish and should be investigated in future studies.

The finding that some vaccinated muskellunge, while protected, may also shed VHSV-IVb into the water column following exposure is troublesome, however, shedding was restricted to a small subset of vaccinated fish that survived the challenge with a relative high virus dose. In the aquatic environment, it is highly unlikely that fish will be exposed to this viral concentration even in VHSV endemic waters [38]. In general, VHSV shedding in fish surviving experimental challenge is of a transient nature [21]. Our data similarly reflects this trend, however, determination and kinetics of shedding kinetics in vaccinated and challenged fish should be ascertained. Unfortunately, all other studies that described vaccine development against VHSV never addressed shedding as a measure of post-challenge vaccine evaluation [10, 15, 16, 26–29].

Herein we have demonstrated that the pVHSivb-G plasmid can elicit significant protection against VHSV-IVb in several species. Though salmonids are less susceptible to genotype IVb, obtaining a protective vaccine will provide researchers and managers with a potent tool that can be used to limit transmission of VHSV-IVb. Most recently, we provided evidence that vaccinated muskellunge can significantly minimize mortalities in cohabitating naïve fish upon experimental challenge, a phenomenon which mimics the concept of herd immunity in terrestrial animals [20]. By vaccinating the millions of propagated salmonid fry and fingerlings prior to stocking, the Great Lakes hatchery system could be used to elicit a large-scale “herd immunity” response. With an overall decrease in the number of naïve-susceptible individuals being stocked, there would be a corresponding decreased viral transmission and an indirect protective effect to other naïve individuals.

## Conclusions

In conclusion, the results we have obtained and the vaccination model we have developed can be used as a first step toward providing managers with tool to stop VHSV dissemination and associated losses. Additionally, the development of an efficacious vaccine preparation allows us to more thoroughly study the nature of immune response in teleosts against serious pathogens.

## Additional files

Additional file 1: Cumulative mortality data for vaccination trials of muskellunge and three salmonid species.ᅟ(XLSX 14 kb)
Additional file 2: Anti-VHSV antibody level in immunized muskellunge pre and post VHSV-IVb challenge.ᅟ(XLSX 12 kb)

## Abbreviations

BNT-1, 2: Brown trout trials
CMV: Cytomegalovirus promoter
IM: Intramuscular
IP: Intraperitoneal
LAT-1, 2: Lake trout trials
MUS-1, 2, 3: Muskellunge trials
OD: Optical density
PV: Post-vaccination
pVHSivb-G: DNA plasmid containing VHSV-IVb glycoprotein gene
RBT-1,2: Rainbow trout trials
RPS: Relative percent survival
RT-qPCR: Quantitative reverse transcription polymerase chain reaction
VPA: Viral plaque assay
